# Annotations

**Published:** 1903-11-07

**Authors:** 


### Annotations.

### The Workhouse and the Gaol.

A disquieting fact is brought out by the recently published report of the Commissioners of Prisons. This is that many people prefer the gaol to the workhouse; so much so that they would rather be taken up as vagrants than go into the workhouse, while those who have taken shelter in the latter are guilty of some offence against the discipline of the establishment in order to be sent to prison, where they receive, according to the report, "better treatment, greater kindness, better food, and more humane conditions." Whether this proves that our prisons are too good or our workhouses too bad for their occupants cannot be decided without personal knowledge of both. Possibly the fact that our prisons are under a central control, so that the conditions are the same in all, while those of our workhouses are more variable, according to the ideals which may prevail with the local authorities, may account for the preference. The man who goes to prison knows, after his first experience, just what to expect, while a new workhouse is more or less of an experiment. Severe management in the workhouse seems invariably to fill the prisons. The governor of one prison, much patronised by the vagrant class, thinks that the task of oakum-picking which the casual pauper is expected to do in the local workhouse, is "an impossible task, even for an experienced oakum-picker," and believes that many of those who refuse to perform it are "genuine working men looking for employment." We do not, for our part, think that a conviction for vagrancy increases the genuine working man's chances of respectable employment, but nevertheless it is a pity that if a man is honest and respectable these qualities should be discouraged by too great harshness in the workhouse. We are told also that when a man who has refused to perform his task in the workhouse is sent to prison for contumacy, there is always the chance that the medical officer of the prison may certify that the prisoner is unfit for the labour, the refusal to perform which, at the workhouse, has resulted in imprisonment. There will always be found casual paupers ready to take that chance, but in any case, it would be just that, before being imprisoned for not doing the work assigned to him, a pauper should be examined in order to make sure that he is physically fit for it. Although he is not, he might be able to do some lighter work. There is surely a mean discoverable between over-work and idleness, and our poor-law system should be sufficiently elastic to permit of paupers being classed, as to the work required of them, according to their strength and fitness. It would be worth while to strain a point to keep our poor from the temptation which seems now to surround them. When the prison is preferred to the workhouse, it tempts to the belief that honesty is not always the best policy.

### Quackery and the Clergy.

An exhaustive study of the annals of human impudence, if such were possible, would no doubt bring to light a large number of curious and interesting examples, which might admit of classification under various heads, and to which quackery would certainly furnish a very large contingent. We question whether all the earlier devices of this craft would not sink into insignificance before one which was described by Sir Edward Fry in the *Times* of October 30th. He forwarded for publication a letter which had been addressed to a Bishop by a limited company describing themselves as "progressive chemists," but whose name and address we shall not promote the success of their devices by publishing. The letter asked his lordship's approval of another letter, which the writers designed to send to the clergy generally, and in which the recipients were asked secretly to furnish lists of the gouty persons within their parishes, who were then to receive puffs of an alleged remedy in the shape of a quack medicine. In the event of a purchase being made by any person so indicated as gouty by his clergyman, or even by any other person in the same locality, the clergyman was to receive a secret commission on the amount; and the clergy were also invited to "take a more active part" in the matter by the dissemination of "leaflets" and other contrivances. Sir Edward Fry appears to be honestly indignant with the persons by whom this arrangement was proposed, and he declares it to be "shocking" that the clergy should "be exposed to such temptations and to such insults." No doubt this is so; but the fault is less with those who have been permitted by the public toleration of their frauds and false pretences to regard quackery as a legitimate form of commerce than with the State under which such an impression has been possible. The State, as represented by the revenue department, will take the money of such people, and will give in return for it a stamp which is regarded by thousands of the ignorant as a guarantee for the "genuineness," if not for the curative properties, of the trash which it identifies and protects. If the trade in quack medicines be an honest one, why should not bishops and the clergy participate in its advantages? If it be utterly dishonest, how is it possible to defend the participation of the State? In so far as the duty on quack medicines produces a revenue, every taxpayer in the country is to a certain extent relieved by it; and, if the moral sense of the community is not outraged we do not see why that of the clergy should be. Probably it may be the proposed secrecy of the transaction to which Sir Edward Fry objects, and to which, in all probability, the clergy would object in an equal degree. But we cannot discover the "insult," even in cases where the proposed commission might be a temptation. Every gentleman is the custodian of his own honour; and cases have not been wanting in which laymen, of a social rank to which the word gentleman would be accorded in ordinary parlance—officers in the army, for example, and the younger sons of men of title—have been by no means inactive in recommending particular brands and vintages to their more opulent friends. We do not suppose the poor quack means any harm. The rascality of quackery has never been brought home to him either by law or by public opinion, and he may have been acting for the best according to his lights. If Sir Edward would strive to deprive him of his present legal status, the example thus set might come in time to exert an enlightening influence.

